# BACOM2.0 facilitates absolute normalization and quantification of somatic copy number alterations in heterogeneous tumor

**DOI:** 10.1038/srep13955

**Published:** 2015-09-09

**Authors:** Yi Fu, Guoqiang Yu, Douglas A. Levine, Niya Wang, Ie-Ming Shih, Zhen Zhang, Robert Clarke, Yue Wang

**Affiliations:** 1 Department of Electrical and Computer Engineering, Virginia Polytechnic Institute and State University, Arlington, VA 22203, USA; 2 Department of Surgery, Memorial Sloan-Kettering Cancer Center, New York, NY 10021, USA; 3 Departments of Pathology and Oncology, Johns Hopkins University, Baltimore, MD 21231, USA; 4 Lombardi Comprehensive Cancer Center, Georgetown University, Washington, DC 20057, USA

## Abstract

Most published copy number datasets on solid tumors were obtained from specimens comprised of mixed cell populations, for which the varying tumor-stroma proportions are unknown or unreported. The inability to correct for signal mixing represents a major limitation on the use of these datasets for subsequent analyses, such as discerning deletion types or detecting driver aberrations. We describe the BACOM2.0 method with enhanced accuracy and functionality to normalize copy number signals, detect deletion types, estimate tumor purity, quantify true copy numbers, and calculate average-ploidy value. While BACOM has been validated and used with promising results, subsequent BACOM analysis of the TCGA ovarian cancer dataset found that the estimated average tumor purity was lower than expected. In this report, we first show that this lowered estimate of tumor purity is the combined result of imprecise signal normalization and parameter estimation. Then, we describe effective allele-specific absolute normalization and quantification methods that can enhance BACOM applications in many biological contexts while in the presence of various confounders. Finally, we discuss the advantages of BACOM in relation to alternative approaches. Here we detail this revised computational approach, BACOM2.0, and validate its performance in real and simulated datasets.

Accurate quantification of somatic copy number alterations in cancers is a systematic effort to discover potential cancer-driving genes such as oncogenes and tumor suppressors^1^,^2^. Bayesian Analysis of COpy number Mixtures (BACOM) is a statistically principled and unsupervised method that exploits allele-specific copy number signals to differentiate between homozygous and heterozygous deletions, estimate normal cell fraction, and recover tumor-specific copy number profiles^1^,^3^ (Methods). The type of data used by BACOM2.0 is high-density and allelic-specific DNA copy number profiles acquired by oligonucleotide-based single nucleotide polymorphism (SNP) arrays. For instance, Affymetrix offers several DNA analysis arrays for SNP genotyping and the newest Affymetrix Genome-Wide Human SNP Array 6.0 features 1.8 million genetic markers including more than 906,600 SNPs^3^,^4^. BACOM was tested on two simulated and two prostate cancer datasets, and very promising results, supported by the ground truth and biological plausibility, were obtained. In our subsequent analyses of TCGA samples with BACOM, we confirmed unexpectedly higher average normal cell fractions. Upon closer examination of the interim results of the entire BACOM analytic pipeline, we found that many normal/amplified copy regions or hemi-deletions were misclassified as homo-deletions. This observation explains the suspected overestimation of normal cell fraction, since the normal cell fraction *α* will be overestimated either when non-deletion regions are wrongly used or when *α*_homo_ is applied to hemi-deletions (*α*_homo_ > *α*_hemi_) in the estimation (Methods).

We propose that the increased estimate of the normal cell fraction is the combined result of imprecise signal modeling and normalization, particularly in the presence of copy-neutral loss of heterozygosity (LOH) and partial aneuploidy. For example, if a non-deletion region is firstly misclassified as a deletion due to imprecise signal normalization, it can be further misclassified as homo-deletion in the cases of allelic balance. Moreover, if the value of the allelic correlation coefficient *ρ* is underestimated due to copy-neutral LOH (allelic-imbalance) contamination in normal/allelic-balanced regions, hemi-deletion will then be misclassified as homo-deletion caused by a much reduced signal-to-noise ratio (Methods).

Accurate signal normalization essentially rescales the relative signal intensities on the basis of normal copy regions (diploid reference loci), here termed as absolute normalization^5^,^6^. As the intertwined result of normal cell contamination, copy number aberrations, and tumor aneuploidy, the average ploidy of tumor cells cannot be assumed to be 2N or an integer^7^. While absolute normalization is critical to inferring absolute copy numbers in a tumor sample, the classic normalization procedure based on median-centering of the total probe intensities is problematic^3^,^4^,^8^ because the dominant component of the signal mixture distribution rarely coincides with the normal copy number ‘2’^7^.

We developed an effective scheme to eliminate the loci belonging to the hemi-deletions (with copy number ‘1’) and the allelic-imbalanced regions. Note that in addition to the odd copy number loci, regions with even copy number can also be allelic-imbalanced, and therefore are also removed. Specifically, we use a sliding window centered at a locus to estimate the inter-allele correlation coefficient and remove those loci whose correlation coefficients are lower than an automatically-determined threshold value. The imbalanced allele signals associated with odd copy numbers would produce a sufficiently negative value of *ρ*. Subsequently, a revised Gaussian mixture model is derived solely from the remaining allelic-balanced loci that highlights the normal copy number loci with ‘sparse’ peaks. Tested on many real copy number datasets, we found that the dominant component of the revised Gaussian mixture distribution now corresponds to the normal copy number regions in most cancer types. In the case of significant chromosome instability or partial aneuploidy, it is possible that the dominant component of the revised Gaussian mixture distribution actually corresponds to copy number ‘4’ (or even higher). Thus, we propose to rescale the measured copy number signal intensities using the mode of the dominant component or the component of the revised copy number signal with the lowest mean value. Since such signal normalization is performed in each individual sample and based on the signals of normal copy number regions, BACOM2.0 implements an accurate and absolute normalization^5^. This procedure also eliminates copy-neutral LOH loci and thus can improve the accuracy of estimating *ρ* by using only normal copy loci and subsequently differentiate between hemi- and homo- deletions (Methods).

We further exploit a mathematically-justified scheme to correct for the confounding impact of intratumor heterogeneity on estimating tumor purity^7^,^9^. Though normal cell fraction *α* can hypothetically be estimated using any deletion segments, it can be experimentally and theoretically shown that the value of *α* will likely be overestimated when intratumor heterogeneity occurs in the deletion segment being used. Thus, in the presence of suspected intratumor heterogeneity, only the ‘pure’ deletion segments with homogeneous tumor genotypes should be used to estimate the normal cell fraction. Based on the distribution of *α* estimates across the whole genome, BACOM2.0 calculates the final value of the normal fraction using the 9-percentile of *α* estimates (Methods).

## Results

### Validation on realistic simulations

**Figure 1:**
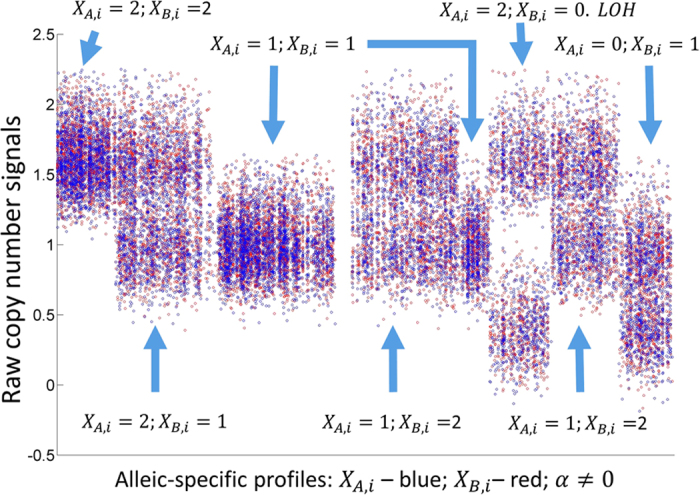
Realistic simulated allelic-specific copy number signals.

We first considered numerical mixtures of simulated normal and cancer copy number profiles across a chromosome region, a situation in which all factors are known and the use of a linear mixture model by equation (1) is valid (Methods). We reconstituted mixed copy number signals by multiplying the simulated cancer copy number profile by the tumor purity percentage in a given heterogeneous sample. Realistic simulations were generated using a specifically selected pair of matched tumor-normal ovarian cancer samples in TCGA, where the tumor somatic copy number profile is approximately normal (allelic-balanced, summed copy number ‘2’, no LOH contamination). After variably dividing the entire region into eight segments, we assigned allelic-specific copy number status to each of the segments ranging from 0 to 3, as specified in Fig. 1. The raw copy number signals (the sum of the two alleles) were produced by mixing 1-α fraction of simulated tumor copy number profile with α fraction of normal copy number profile, as given in equation (1). This simulation represents a highly challenging scenario in which most probe sets were not ‘normal’ but amplified, yet also contained both hemi-deletion and copy-neutral LOH segments.

**Figure 2:**
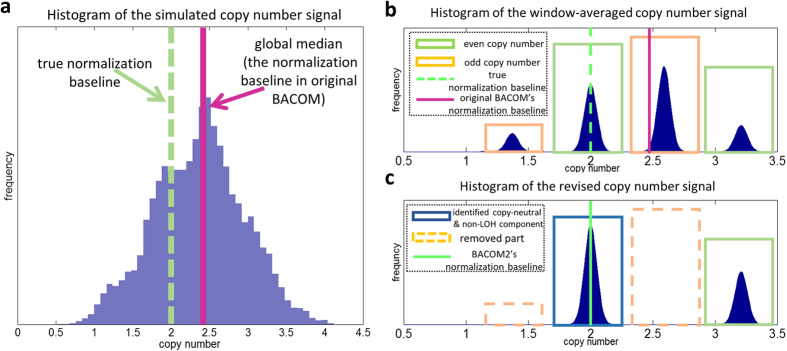
(**a**) Histogram of simulated copy number signals; (**b**) Histogram of preprocessed copy number signals after moving-average; (**c**) ‘revised’ histogram of copy numbers after eliminating allelic-imbalanced regions.

Using the BACOM2.0 analytic pipeline, we first calculated the histogram of the raw copy number signals (Fig. 2a); then we preprocessed the raw copy number signals by a moving-average low-pass filter that significantly reduced the noise effect, and re-calculated the histogram (Fig. 2b); finally we eliminated all allelic-imbalanced loci and generated a revised histogram where the dominant peak correctly coincided with the normal copy number ‘2’ component (Fig. 2c).

With a successful absolute normalization, we first checked the estimated value of the between-allele correlation coefficient ρ, and then recalculated the normal cell fraction α. Based on the comparative estimates given in Table 1, the power of BACOM2.0 is evident since the model parameter estimates are very close to the ground truth when compared with the output obtained using the original BACOM. Additional information on validation design and experimental results is included in the Supplementary Information.

### Analysis of benchmark real copy number data

**Figure 3:**
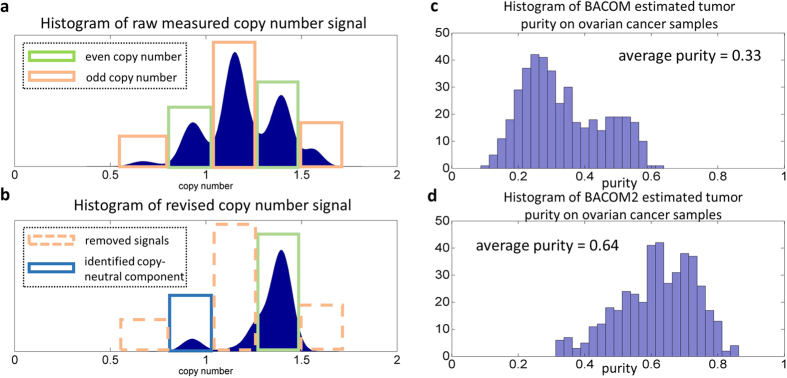
Analysis by BACOM2.0 on the real TCGA ovarian cancer samples. (**a**) Histogram of copy number signals after moving-average operation; (**b**) Histogram of ‘revised’ copy number signals after eliminating allelic-imbalanced loci; (**c**) Histogram of the overall tumor purities estimated by original BACOM from 466 OV samples; (**d**) histogram of the overall tumor purities estimated by BACOM2.0 from 466 OV samples.

We then applied BACOM2.0 to the challenging case of the TCGA ovarian cancer dataset (n = 466 samples). Substantial genomic instability has been well-documented in high-grade ovarian cancers^10^,^11^,^12^. We observed that, in a large number of tumor samples, the dominant component of raw measured copy number histogram does not correspond to the normal copy number ‘2’ but rather to the allele-imbalanced loci (Fig. 3a). This observation suggests the presence of partial aneuploidy in these samples, and highlights the improper use of global mean/median as the normalization baseline^5^.

Using the BACOM2.0 analytic pipeline, we processed the raw measured copy number signals using a moving-average low-pass filter, eliminated all allelic-imbalanced loci, generated a revised histogram, and identified the component of normal copy number ‘2’ (Fig. 3b). With a successful absolute normalization, we estimated tumor purity and tumor-specific copy number profile on each sample. From a comparison between the histogram of tumor purities likely underestimated by the original BACOM from 466 OV samples (Fig. 3c) and the histogram of tumor purities newly estimated by BACOM2.0 from 466 OV samples (Fig. 3d), we can see that BACOM2.0 produced much higher tumor purity estimates (average purity of 64% versus 33%) that are theoretically expected and consistent with the protocol baseline adopted in independent studies (using 50% purity as the threshold to differentiate between high and low tumor purity in three cancer types)^13^,^14^,^15^.

**Figure 4:**
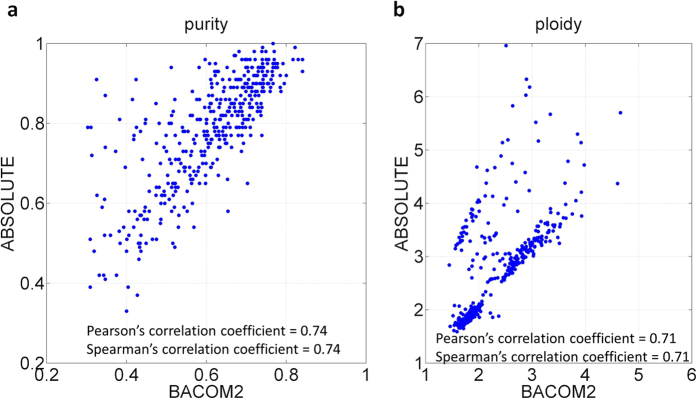
Sample-wise comparison between the estimates of tumor purity and average ploidy by BACOM2.0 and ABSOLUTE on 392 TCGA ovarian cancer samples. (**a**) Scatter plot of tumor purity estimates; (**b**) Scatter plot of tumor ploidy estimates.

Using the same dataset, we further compared the estimates generated by BACOM2.0 with those produced by ABSOLUTE^4^. As a closely relevant method, ABSOLUTE reports the estimates of tumor purity and average ploidy on two TCGA datasets, ovarian cancer (OV) and brain cancer (GBM). With a quality control selection on paired tumor and normal samples, ABSOLUTE analyzed n = 392 tumor samples in the OV dataset. Note that the tumor purity estimates, obtained by ABSOLUTE and used in our comparison, have been partially validated experimentally^4^. The average tumor purity estimates by BACOM2.0 and ABSOLUTE are 64% and 78%, respectively; and the average tumor ploidy estimates by BACOM2.0 and ABSOLUTE are 2.33 and 2.73, respectively. The sample-wise correlation coefficients show that both tumor purity and tumor ploidy estimates by BACOM2.0 correlate well with the estimates by ABSOLUTE (Fig. 4), achieving high correlation coefficients of *r* = 0.74 on purity and *r* = 0.71 on ploidy. On the GBM dataset, the average tumor purity estimates by BACOM2.0 and ABSOLUTE are 59% and 71%, respectively; and the average tumor ploidy estimates by BACOM2.0 and ABSOLUTE are 2.09 and 2.17, respectively. Additional information on additional experimental results (tables and figures) is included in the Supplementary Information.

### Cross-affirmation by expression deconvolution

**Figure 5:**
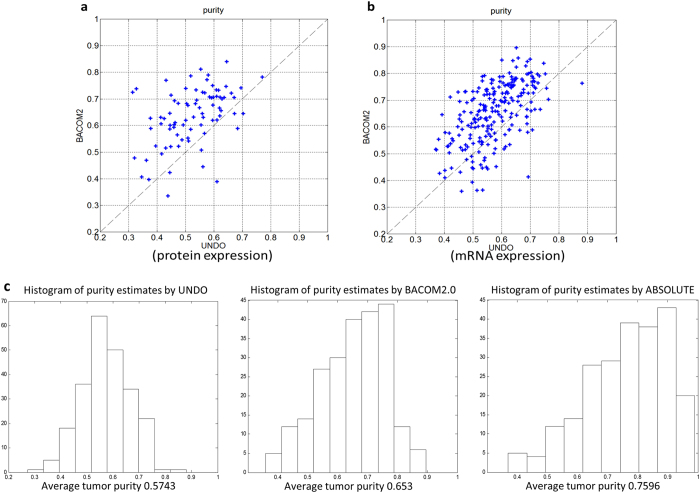
(**a**) Sample-wise correlation between tumor purity estimated by BACOM2.0 using copy number data and by UNDO using protein expression data; and (**b**) Sample-wise correlation between tumor purity estimated by BACOM2.0 using copy number data and by UNDO using gene expression data; (**c**) Histograms of the tumor purity estimates by UNDO using gene expression data, by BACOM2.0 using copy number data, and ABSOLUTE using copy number data; on the same TCGA_OV samples.

In the absence of definite ground truth about the tumor purities in real samples, the validation of a new method for quantifying absolute copy numbers is always problematic. A reasonable alternative is to perform some form of ‘cross’ affirmation by exploiting the ‘orthogonal’ information structures provided by the independent sources related to a common set of nature states^16^. Finally, we compared the tumor purity estimates by BACOM2.0 with the estimates by an independent method (called UNDO) that deconvoluted the mixed gene/protein expression profiles of tumor and stroma cells acquired from the same TCGA OV samples^17^. Using the UNDO software, we analyzed the tumor samples with consistent purity estimates by both BACOM2.0 and ABSOLUTE. The experimental result shows that the tumor purity estimates by BACOM2.0 (based on copy number data) correlates well with the estimates by UNDO (based on gene expression data and protein expression data^18^), consistently achieving a strong average ‘cross’ correlation coefficient of 0.5 ~ 0.6 in multiple runs (Fig. 5a,b). The protein expression data on TCGA ovarian cancer samples were acquired using state-of-the-art mass spectrometry technologies by CPTAC (Clinical Proteomic Tumor Analysis Consortium), to define an integrated proteogenomic landscape and identify factors associated with homologous recombination deficiency (HRD)^18^. These tumor samples were previously characterized by TCGA^19^, and key findings were the initial identification of transcriptional signatures associated with survival and the role of BRCA1/2 and CCNE1 aberrations, and an association between HRD and susceptibility to PARP inhibitors and improved survival. The dataset provides quantitative measurements for 10,030 proteins across 122 tumors. To identify a proteomic signature of HRD that could potentially be used to stratify patients for clinical trials^20^, tumors were selected for putative HRD, defined by the presence of germline or somatic BRCA1 or BRCA2 mutations, BRCA1 promoter methylation, or homozygous deletion of PTEN^21^. Isobaric peptide labeling (iTRAQ) for quantitation in conjunction with extensive fractionation and high resolution reversed phase liquid chromatography and high-resolution tandem mass spectrometry was used for proteomics measurements to provide high depth of coverage for peptide and protein identification. Moreover, our experimental result on TCGA-OV samples shows that the mean of the estimated tumor purities obtained by BACOM2.0 is much closer to the result obtained by UNDO than the result obtained by ABSOLUTE (Fig. 5c)^17^.

The imperfect ‘cross’ correlation between the tumor purity estimates by BACOM2.0 and UNDO is expected and can be justified^17^ because the two methods use different molecular data types where copy number values are always ‘2’ across all normal cells (e.g., stroma, T-cells, monocytes) while gene expression values are cell type specific. Multiple gene expression profiles correspond to various normal cells and copy number values are generally ‘static’, while gene expression values are intrinsically ‘dynamic’. For example, in the recent supervised deconvolution work called ESTIMATE by Yoshihara *et al.*^22^, to obtain a high correlation between the tumor purity estimates derived from copy number and gene expression data, a nonlinear regression function was used to map the ‘score’ by ESTIMATE^22^ to the estimate by ABSOLUTE^4^. Though a higher correlation was obtained and validated on multiple datasets after such nonlinear mapping, it is somewhat ‘indirect’.

### Open-source software packages for BACOM2.0 and original BACOM

An open-source BACOM2.0 software is developed and is publically available at https://code.google.com/p/bacom2/.

To better serve the research community, we previously developed a cross-platform and open-source BACOM Java application, which implements the entire pipeline of copy number change analysis for heterogeneous cancer tissues including extraction of raw copy number signals from CEL files, classic data normalization, identification of AB loci, copy number detection and segmentation, probe sets annotation, differentiation of deletion types, estimation of the normal tissue fraction, and correction of normal tissue contamination. Interested readers can freely download the software and source code at http://www.cbil.ece.vt.edu/software.htm,https://code.google.com/p/aisaic/

To take advantage of many widely used packages in R, we also implemented an R interface, bacomR, that enables users to smoothly incorporate BACOM into their routine copy number analysis pipeline or to integrate BACOM with other R or Bioconductor packages. Users can use their preferred methods to perform routine tasks such as array normalization and DNA copy number segmentation and estimation, while using the newly added BACOM to estimate the normal cell fraction and subsequently recover the true copy number profiles in pure cancer cells.

## Discussion

In this report, we describe methods with enhanced ability to more accurately detect deletion types, estimate normal cell fraction, and quantify true copy numbers in tumor cells. We achieved these objectives by introducing a more comprehensive signal modeling and absolute normalization scheme^5^. BACOM2.0 corrects and extends the BACOM method^3^, and offers several attractive features including (1) performing absolute normalization by identifying the normal copy number component in a ‘revised’ Gaussian mixture histogram; (2) estimating signal models and their parameter values after eliminating significant confounding factors; (3) calculating the overall normal cell fraction (or tumor purity) with a correction for potential intratumor heterogeneity; and (4) adjusting the effect of copy number signal saturation. While the principal application here involves SNP arrays, our methodology is also applicable to sequence derived copy number data^1^.

Fundamental to the success of our approach is the rigorous signal modeling and absolute normalization. In the presence of both normal cell contamination and tumor aneuploidy, with proper sample quality control^6^, absolute normalization can be done separately (or iteratively) from tumor purity/ploidy estimation^5^. We expect BACOM2.0 to be a useful tool for analyzing copy number data in heterogeneous tumor samples^1^,^2^, and as a complement to existing methods^4^,^9^.

We acknowledge that the absolute normalization step in BACOM2.0 may fail for highly aneuploid tumors. When no or very few regions are present at normal copy number, while this difficulty is mathematically expected, it has not been discussed effectively in the literature^4^,^7^,^23^,^24^. In BACOM2.0, we have taken several approaches to address this problem. Absolute normalization can be performed at both the chromosome-wide and the genome-wide scales and the results are then compared. Based on a survey across multiple cancer types, see Fig. 3c in^4^, most cancer types have an average ploidy of ‘2’. Thus, even for highly aneuploid tumors, BACOM2.0 may still perform well when there are sufficient regions at normal copy number in some chromosomes. When the normalization results obtained from the chromosome-wide and the genomic-wide scales are inconsistent, BACOM2.0 adopts a chromosome-wide scale analyses on subsequent tasks. Moreover, the window-based moving-average and elimination of allelic-imbalanced loci in BACOM2.0 can also ease the impact of aneuploidy.

We also acknowledge that the tumor purity estimation step in BACOM2.0 may fail when no or few homogeneous deletions are present. The impact of intratumor heterogeneity on copy number analysis has been previously acknowledged^25^. While accurate estimation of tumor purity in the presence of significant intratumor heterogeneity is difficult, we have also incorporated novel approaches to address this problem in BACOM2.0. Specifically, tumor purity is estimated segment by segment, with varying lengths, in deletion regions. Assume that the absolute normalization is correctly achieved, since there will be only a limited number of possible combinations among deletion-subclones in any deletion segment, BACOM2.0 performs well by calculating the overall tumor purity using the 9-percentile of segment-wide purity estimates across the whole genome.

In relation to previous work, the concept of using allele-specific information for analyzing copy number data is shared by others^23^, and was further developed by Rasmussen *et al.*^7^ for exploratory data visualization in conjunction with visual inspection of aneuploidy and tumor heterogeneity. There is also some similarity between our objectives and others in cancer copy number restoration and tumor purity estimation. The major limitations of the approach by Yuan *et al.*^26^ are that it requires matched genomic and histopathological image data and relies heavily on image quality (coarse H&E staining, artifacts, batch effects). ABSOLUTE, which was developed by Carter *et al.*^4^, is supported by an elegant yet complex mathematical framework and can select the most likely combination of estimated tumor purity and ploidy by integrating copy number data and supervised learning. However, the cornerstone system of equations is underdetermined and various heuristics cannot guarantee a unique and correct solution^9^. In the presence of more than one tumor subclone, the restored copy number signals are not necessarily all integer values. Thus, using the highest likelihood of producing all integer signals to select the most likely solution from the multiple solutions to the systems of equations may be problematic^9^. For example, in the presence of more than one tumor subclone, the solution with the highest likelihood of producing all integer copy numbers would be theoretical wrong because single *α* corrected signals (removal of normal contamination) should not lead to an all integer copy number estimate. In contrast, BACOM2.0 adopted a divide-and-conquer strategy by sequentially performing absolute normalization, purity estimation, copy number quantification, and average-ploidy estimation. PurityEst proposed by Su *et al.*^15^ estimates normal cell fraction using single-nucleotide variants but not original sequence reads. The formulation does not explicitly consider effects of copy number gains/losses and may bias tumor purity estimation. Moreover, PuriryEst^15^, THetA^9^, and AbsCN-seq^27^ rely on next-generation sequencing data, thus may not be applicable to existing copy number data acquired using more classic methods such as SNP arrays.

Since BACOM2.0 is supported by a well-grounded and unambiguous statistical framework, we foresee a variety of extensions to the concepts and strategies here. Regarding the detection of allelic-imbalanced loci, a good alternative to allelic correlation coefficient is the B allele frequency ratio^5^,^6^,^24^. When there are multiple deletion segments across a genome, the distribution of *α* estimates merits further study since it may indicate the presence of intratumor heterogeneity defined by subclone copy number aberrations. In our experimental studies, we have found that some tumor samples exhibit a wide-spread distribution of *α* values across different segments, consistent with the observation in other studies specifically addressing intratumor heterogeneity^23^. Moreover, with further development, localized chromosomal ploidy can be detected instead of average tumor ploidy^24^.

While a significant sample-wise correlation between the tumor purity estimates by BACOM2.0 and ABSOLUTE has been observed, further investigation into the discrepancy between the average tumor purity estimates by the two methods would be interesting, given the fact that no definite ground truth is available. For example, TCGA used 60 ~ 80% tumor purity as the threshold to select tumor samples, while a protocol baseline of 50% tumor purity was adopted to differentiate between high and low tumor purity in three cancer types^13^,^14^,^15^, and yet rather poor correlations were reported between the estimates by ABSOLUTE or ESTIMATE and the histological analysis probably due to miscount of infiltrating immune cells in pathological examinations^22^. Another limitation of this report is the complete reliance on *in silico* and publically available datasets. To strengthen the work, future studies could perform SNP array analysis on the DNA extracted from a set of tumours or a handful of cell lines (which typically possess multiple gross aneuploidies due to immortalization and passaging), with subsequent validation of the copy-number changes identified by BACOM2.0 confirmed by digital PCR. Nevertheless, the data we have used here is publically available, allowing others to fully replicate our findings.

## Methods

In this study, the data used is high-density and allelic-specific DNA copy number profiles acquired by oligonucleotide-based single nucleotide polymorphism (SNP) arrays. The TCGA data sets were mainly acquired using Affymetrix DNA analysis arrays for SNP genotyping, *e.g.*, Affymetrix Genome-Wide Human SNP Array 6.0 with 1.8 million genetic markers including more than 906,600 SNPs^1^,^4^. The DNA copy number associated with a somatic cell or cell type is called the genuine copy number and is assumed to be integers^28^. In complex tissues such as a tumor sample, the DNA copy number would be a weighted average of the genuine copy numbers associated with tumor and stromal cells in that sample and is assumed to be real numbers^1^,^3^. The existence of copy-neutral loss of heterozygosity (LOH) and partial aneuploidy presents an additional layer of complexity to dissect mixed copy number signals^4^. To accurately estimate DNA copy numbers in tumor cells, two major analytic tasks are (1) absolute signal normalization and (2) tumor purity estimation. BACOM2.0 is developed to facilitate absolute normalization and quantification of somatic copy number alterations in heterogeneous tumor.

### BACOM overview^1^,^3^

In a heterogeneous tumor sample, the measured copy number signal is a mixture of the signals from both normal and cancer cells,
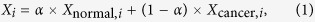
where *X*_*i*_ is the observed copy number signal at locus *i*, *α* is the unknown fraction of normal cells, *X*_normal,*i*_ and *X*_cancer,*i*_ are the latent copy number signals in normal and cancer cells at locus *i*, respectively. Let *X*_*A*,*i*_ and *X*_*B*,*i*_ be the allele-specific copy number signals, *X*_*i*_ = *X*_*A*,*i*_ + *X*_*B*,*i*_ are assumed to be independently and identically distributed random variables following a normal distribution 
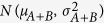
 whose mean *μ*_*A*+*B*_ and variance 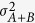 can be readily estimated by the sample averages. Allele-specific analyses are focused on the deletion regions with distinct genotypes^3^. Types of deletions are detected by a model-based Bayesian hypothesis testing. Specifically, BACOM uses a novel summary statistic,
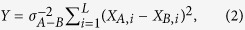


where 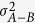 is the variance of *X*_*A*_,_*i*_ − *X*_*B*_,_*i*_ in a length-*L* deletion region. It has been shown that under homo-deletion, *Y* follows an *L* degrees of freedom standard 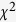 distribution, given by
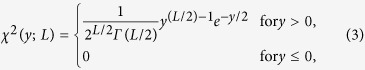
and under hemi-deletion, *Y* follows an *L* degrees of freedom noncentral 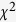 distribution, given by
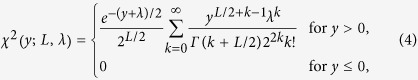
where 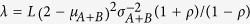, *ρ* is the genuine allelic correlation coefficient between *X*_*A*,*i*_ and *X*_*B*,*i*_,
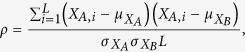
and Γ denotes the Gamma function. Since for a deletion region, we have
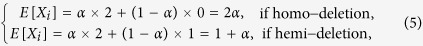
then, the average normal cell fraction 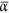 across the whole genome can be estimated^3^, as well as cancer-specific copy number profiles, given by
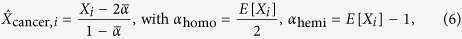


### Problem diagnosis

More detailed reasoning on the root causes of the underestimated tumor purity by BACOM method are given in Supplementary Information.

### Allele-specific absolute normalization

Let us consider histogram modeling of genome-wide copy number signals. Based on the underlying signal characteristics, we adopt a mixture of *K* Gaussian distributions^5^, given by
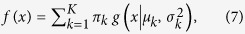
where *π*_*k*_ is the relative proportion of the *k-*th copy number component and *g*(.|.) is the Gaussian kernel with 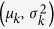 being the mean and variance. Such mixtures can be estimated from the observed histogram or raw data based on the maximum likelihood principle^29^. In BACOM2.0, the estimation was implemented by the EM (expectation-maximization) algorithm in which its E-step is also called soft clustering^30^. However, our experimental studies on real tumor data confirmed that the component mean with the largest *π*_*k*_ does not always correspond to the mean of normal copy regions and cannot serve as the baseline for absolute normalization. We have also observed that the largest component(s) often resides within the neighborhood of the normal copy component (with copy number ‘1’, ‘3’, ‘4’, etc.).

### Accuracy of estimating *ρ*

It can be shown that the imbalanced allele signals associated with odd copy numbers would produce a sufficiently negative value of *ρ*, given by (in the case of copy number ‘3’)
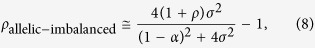
where *σ*^2^ is the variance of noise and *ρ* is the genuine inter-allele correlation coefficient. Furthermore, it can be shown that copy-neutral LOH contamination will result in an inaccurate estimate of *ρ*, given by
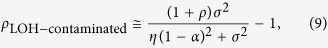
where *η* is the percentage of copy-neutral LOH contamination. Thus, BACOM2.0 includes an accurate estimation of the allelic correlation coefficient *ρ* (related to model parameter λ in defining hemi-deletion summery statistic) that was often underestimated due to copy-neutral LOH contamination. Again, by excluding copy-neutral LOH loci and identifying the correct normal copy regions we can now obtain a more accurate estimate of allelic correlation coefficient *ρ*.

### Calibration of allele signal crosstalk

We also calibrated allele signal crosstalk and saturation effects. Theoretically, signal crosstalk from the probes that differ only in one SNP adds positive bias to the copy number estimate that could lead to an overestimation of normal cell fraction by equation (6). Allelic crosstalk also biases the estimate of the allele correlation coefficient *ρ*. Concerning copy number signal saturation using SNP arrays, we adopted a similar linearization strategy used by ABSOLUTE^4^.

### Correction of confounding intratumor heterogeneity

With an accurate absolute signal normalization, the normal cell fraction *α* (or tumor purity 1-*α*) can be estimated segment-by-segment, using the scheme discussed in the main text or elsewhere^1^,^3^. When tumor cells are homogeneous, the values of *α* estimated from different segments is expected to be very close. However, due to potentially significant intratumor heterogeneity^7^,^9^,^23^, we have observed that in some real copy number datasets the distributions of segment-based purity estimates *α* are scattered^3^. This intratumor heterogeneity presents an additional layer of complexity to estimating tumor purity^4^. It can be theoretically illustrated that the normal cell fraction *α* will be overestimated when significant intratumor heterogeneity exists, since within a segment there may only be a fraction of tumor cells with deletions.

For example, assume that there are only two deletion-type subclones in the tumor cells. In a copy number segment, there are a total of 5 copy number combinations among normal cell, tumor clone 1, and tumor clone 2: (1) normal, hemi, hemi; (2) normal, homo, homo; (3) normal, hemi, normal; (4) normal, homo, normal; and (5) normal, hemi, homo. Only the first 2 cases represent a homogeneous tumor subpopulation that can be well-handled by the existing scheme used in the original BACOM. For cases 3 and 4, even an overall ‘deletion’ is correctly detected, the deletion type is leaning to homo-deletion type due to normal copy number fraction in a tumor subpopulation. Thus, normal cell fraction *α* will be overestimated, due to an imprecisely higher value of *E*[*X*_*i*_] and imprecise use of *α*_homo_. For case 5, there is a high probability that normal cell fraction *α* will be overestimated, once again due to potential misclassification as a pure homo-deletion and the imprecise use of *α*_homo_.

In BACOM2.0 we use empirically the 9-percentile of the genome-wide *α* estimates to calculate the final estimate of sample-specific tumor purity. Rather than using the mean of ‘local’ purity estimates over genome-wide deletion segments, we use the 9-percentile of ‘local’ purity estimate distribution to determine the overall tumor purity in a sample. The rationale behind this choice is twofold. Firstly, there are 5 possible combinations of 2 deletion types in an overall deletion segment. Only 2 of these 5 scenarios can produce an accurate tumor purity estimate. Since purity estimation on homo-deletion segment is less reliable, due to the potential confusion between ‘normal’ and ‘homo-deletion’, the 9-percentile of ‘local’ purity estimate distribution should capture the most reliable and accurate overall tumor purity estimates that are associated with homogeneous hemi-deletion segments. Secondly, the tumor purity estimation using the 9-percentile of ‘local’ purity estimate distribution on TCGA datasets has produced consistent results that correlated well with the estimates obtained by the benchmark method ABSOLUTE. Nevertheless, we acknowledge that this choice is empirical (based on our experience with TCGA datasets) and alternative schemes should be further studied.

### BACOM2.0 flowchart

**Figure 6:**
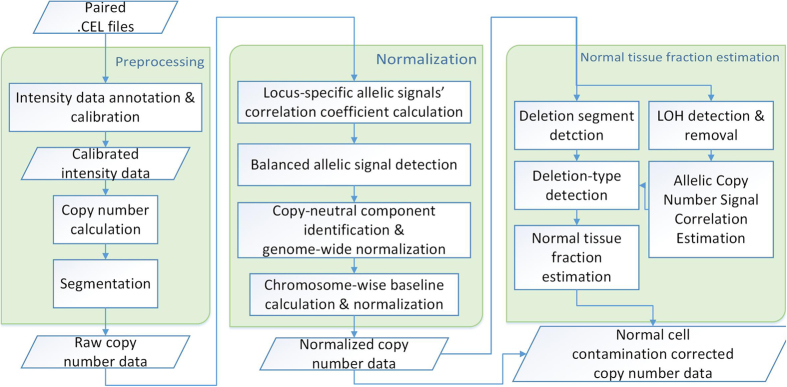
Analytic pipeline of BACOM2.0: schematic flowchart.

More information on BACOM2.0 method and algorithm, summarized in Fig. 6, is included in Supplementary Information.

## Additional Information

**How to cite this article**: Fu, Y. *et al.* BACOM2.0 facilitates absolute normalization and quantification of somatic copy number alterations in heterogeneous tumor. *Sci. Rep.* **5**, 13955; doi: 10.1038/srep13955 (2015).

## Supplementary Material

Supplementary Information

## Figures and Tables

**Table 1:** Comparative parameter estimates by BACOM and BACOM 2.0.

**Parameter**	**Ground truth**	**BACOM**	**BACOM2.0**
*ρ*	−0.042	−0.714	−0.063
*α*	40%	79%	39%
