# Supplementary material for: BACOM2.0 facilitates absolute normalization and quantification of somatic copy number alterations in heterogeneous tumor
Source: Sci Rep. 2015 Sep 9;5:13955. doi: 10.1038/srep13955 (PMC4563570; doi:10.1038/srep13955)
Supplement: Supplementary Information [file srep13955-s1.pdf]

Supplementary Information

BACOM 2.0 facilitates absolute normalization and quantification of somatic copy number alterations in heterogeneous tumor

Yi Fu[1], Guoqiang Yu[1], Douglas A. Levine[2], Niya Wang[1],
Ie-Ming Shih[3], Zhen Zhang[3], Robert Clarke[4], and Yue Wang[1]

[1]Department of Electrical and Computer Engineering, Virginia Polytechnic Institute and State University, Arlington, VA 22203, USA;

[2]Department of Surgery, Memorial Sloan-Kettering Cancer Center, New York, NY 10021, USA;

[3]Departments of Pathology and Oncology, Johns Hopkins University, Baltimore, MD 21231, USA;

[4]Lombardi Comprehensive Cancer Center, Georgetown University, Washington, DC 20057, USA

Contents

1 Relevant problems in BACOM 1

1.1 Inaccurate signal normalization 1

1.2 Misclassification of deletion types 2

2 Data preprocessing and signal intensity calibration 3

2.1 Reading raw data 3

2.2 Observed signal intensity modeling 3

2.3 Attenuation correction 5

2.4 Segmentation 5

3 Model estimation and absolute normalization 5

3.1 Inter-allele correlation 5

3.2 Identifying copy-neutral loci and genome-wide normalization 6

3.3 Chromosome-wise finer normalization 7

4 Simulation studies 8

5 Analyses of real copy number data 12

5.1 BACOM 2.0 versus BACOM 12

5.2 BACOM 2.0 versus ABSOLUTE 14

5.3 Cross-affirmation by deconvolution on mRNA/protein expression data 16

6 Discussion 18

1 Relevant problems in BACOM

In our independent analyses of TCGA samples using BACOM, we confirmed unexpectedly high average normal cell fractions. Upon closer examination of the interim results of the entire BACOM analytic pipeline, we found that many normal/amplified copy regions and hemi-deletions were misclassified as homo-deletions.

1.1 Inaccurate signal normalization

Accurate signal normalization essentially rescales the relative signal intensities on the basis of normal copy regions (diploid reference loci), here termed as absolute normalization [1,2]. As the intertwined result of normal cell contamination, copy number aberrations, and tumor aneuploidy, the average ploidy of tumor cells cannot be assumed to be either 2N or an integer [3]. Though absolute normalization is critical to inferring absolute copy numbers in a tumor sample, the classic normalization procedure based on median-centering of the total probe intensities is problematic [4-6], since the dominant component of the intensity mixture distribution rarely coincides with the normal copy number '2' (Rasmussen, et al., 2011). Unfortunately, original BACOM adopted the classic normalization scheme that is clearly inaccurate.

Consider one typical example. If signal normalization is imprecisely done using a biased scale, e.g., a true copy-neutral segment (corresponding to copy number '2') is misclassified as 'deletion'. Theoretically, it will be further detected as 'homo-deletion' because of the inability of the summary statistic, under the central χ^2 distribution

$$Y = \sigma_{A-B}^{-2} \sum_{i=1}^{L} (X_{A,i} - X_{B,i})^2,$$

to distinguish between copy-neutral segment and homo-deletion segment [6]. Accordingly, the normal cell fraction α wrongly estimated using a copy-neutral segment would be higher than the true value (assuming that tumor cell has copy number '3'):

$$\hat{\alpha} = \frac{E[X_i]}{2} = \frac{4}{3-\alpha} \times \frac{1}{2} = \alpha + \frac{(1-\alpha)(2-\alpha)}{3-\alpha} > \alpha,$$

where the observed signal mean $E[X_i] = 3-\alpha$ is used for normalization and

$$E[X=2]=\frac{4}{3-\alpha}<2,\ \alpha\in[0,1),$$

is firstly misclassified as 'deletion'.

1.2 Misclassification of deletion types

Once a deletion segment is identified, correct classification of deletion types (hemi- versus homo-) is a critical step in the BACOM methodology [6]. Our experimental results indicate that misclassification of deletion types occurs and is mainly caused by the inaccurate estimation of inter-allele correlation coefficient due to both LOH contamination and inter-allele crosstalk. Theoretically, without a crosstalk effect or a crosstalk effect can be 'calibrated', an inter-allele correlation coefficient of near 'zero' is expected. Since which homologous chromosome an allelic signal belongs to is often unknown, calculation of the inter-allele correlation coefficient can only be done over 'allele-balanced' loci. Original BACOM estimates the inter-allele correlation coefficient using AB-genotyped copy-neutral sites that contain both allele-balanced and allele-imbalanced loci.

Consider a few examples here. If an allele-imbalanced segment (assuming copy number '3') is used in the calculation (due to inaccurate signal normalization), the inter-allele correlation coefficient would be biased (negative):

$$\hat{\rho}=\frac{E\left[(X_A-\mu_A)(X_B-\mu_B)\right]}{\sigma_A\sigma_B}\cong-1+\frac{4(1+\rho_0)\sigma^2}{(1-\alpha)^2+4\sigma^2},$$

where μ_A and μ_B are the means of allelic copy numbers, σ_A and σ_B are the standard deviations ($=\sigma$), and ρ_0 is the genuine value of inter-allele correlation coefficient. Let $\rho_0=0$, $\alpha=40\%$, and $\sigma=0.2$, the estimation would be $\hat{\rho}\cong-0.69$, much lower than the genuine value $\rho_0=0$. Moreover, if a true copy-neutral or allele-balanced segment is contaminated with a significant portion of copy-neutral LOH loci (e.g., high frequency of copy-neutral LOH has been observed in high-grade ovarian cancer samples in TCGA), denote η as the portion of copy-neutral LOH sites in the copy-neutral component the inter-allele correlation coefficient would be biased (negative):

$$\hat{\rho}\cong-1+\frac{(\rho_0+1)\sigma^2}{\eta(1-\alpha)^2+\sigma^2},$$

where η is the fraction of copy-neutral LOH sites in the segment. Let $\eta = 20\%$ and keep the above parameter settings, the estimation would be $\hat{\rho} \cong -0.64$, once again much lower than the genuine value $\rho_0 = 0$.

When the inter-allele correlation coefficient is underestimated, the resulting summary statistic would also be underestimated [6]:

$$Y = \frac{1+\hat{\rho}}{1-\hat{\rho}} \sigma_{A+B}^{-2} \sum_{i=1}^{L} (X_{A,i} - X_{B,i})^2 < \frac{1+\rho_0}{1-\rho_0} \sigma_{A+B}^{-2} \sum_{i=1}^{L} (X_{A,i} - X_{B,i})^2.$$

Furthermore, since the classification of deletion type in BACOM is based on hypothesis testing of Y under non-central (hemi-deletion) versus central (homo-deletion) χ^2 distributions, an underestimate of $L+\lambda$ would decrease the signal-to-noise ratio (differential power) between the two hypotheses, where $\lambda = L(2-\mu_{A+B})^2 \sigma_{A+B}^{-2} (1+\rho)/(1-\rho)$. As explained in the main text, a misclassification of hemi-deletion to homo-deletion would overestimate the normal cell fraction α because it is always true that

$$\hat{\alpha}_{\text{homo}} = E[X_i]/2 > \hat{\alpha}_{\text{hemi}} = E[X_i]-1.$$

2 Data preprocessing and signal intensity calibration

2.1 Reading raw data

BACOM 2.0 uses allelic information (allelic summary statistics, allele- balance/imbalance, inter-allele correlation) to determine deletion type and subsequent analyses. Reading raw data from '.CEL' files in BACOM 2.0 is performed as in the original BACOM [6,7]. The platform library file provided by Affymetrix is used to extract the optical scanned intensity value for each probe. Probes' chromosome IDs, locations, and probe set information are then annotated with the standard annotation library file.

2.2 Observed signal intensity modeling

In the observed signal intensity, two major confounding factors are inter-allele crosstalk and positive noise offset that can potentially bias the quantification of true copy numbers (**Supplementary Figure S1**) [8,9].

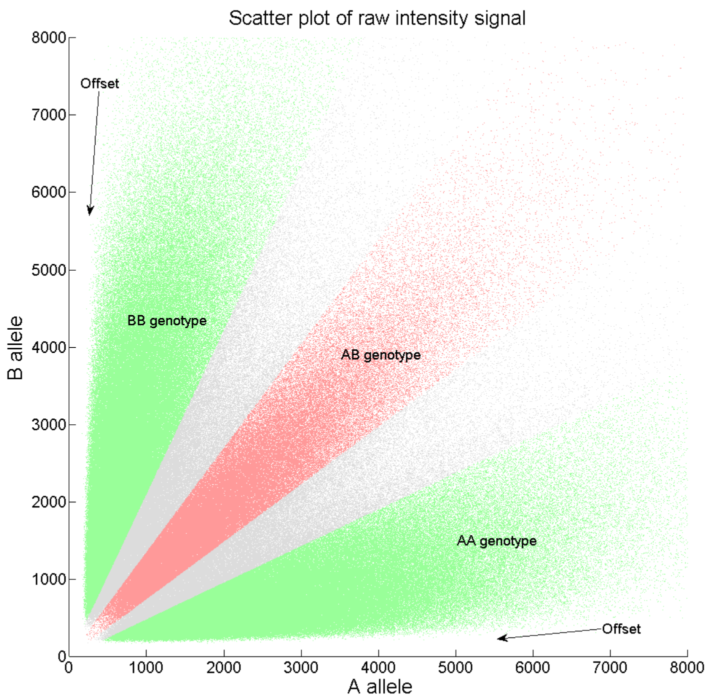

Supplementary Figure S1. Scatter-plot of two allelic signal intensities from a real array sample. The positive offset in signal intensity is marked.

In BACOM 2.0, we use a linear regression model to correct for both signal crosstalk and noise offset, based solely on AA/BB-genotyped probes. For example, for AA-genotype ($X_{A,i}=2$, $X_{B,i}=0$), the observed signal intensity $\hat{X}$ can be modeled as (assuming signal normalization is correctly done):

$$\hat{X}_{B,i}=\theta X_{A,i}+X_{B,i}+\left(1-\theta\right)\gamma+\varepsilon=2\theta+\left(1-\theta\right)\gamma+\varepsilon,$$

where θ represents the crosstalk effect, γ represents the noise offset, and ε represents the white noise. A model fitting procedure can be performed to estimate the values of θ and γ. Our experimental results indicate that θ ranges from 0.1 to 0.15 and γ ranges from about 100 to 400. With accurately estimated θ and γ, signal correction can be done principally by

$$\begin{bmatrix} X_{A,i} \\ X_{B,i} \end{bmatrix} \triangleq \begin{bmatrix} 1 & \theta \\ \theta & 1 \end{bmatrix}^{-1} \begin{bmatrix} \hat{X}_{A,i}-\gamma \\ \hat{X}_{B,i}-\gamma \end{bmatrix}.$$

2.3 Attenuation correction

Signal attenuation is widely observed and is mainly caused by the nonlinearity of optical intensity associated with large copy number alterations [4,8]. In BACOM 2.0, we use the Langmuir isothermal absorption model to correct for signal attenuation [4,9].

2.4 Segmentation

In BACOM 2.0, absolute signal normalization is performed based on the identified signal mean of individual copy-neutral segments (corresponding to true copy number '2') [6,7]. Slight different from original BACOM, here we perform signal segmentation before signal normalization [10]. This rearrangement makes no difference in the analytic outcomes, since the segmentation scheme is invariant to the linear scaling used in signal normalization [6,10].

3 Model estimation and absolute normalization

To perform accurate absolute normalization, identification of copy-neutral or normal copy number loci is a critical step. In BACOM 2.0, this is done by removing allele-imbalanced loci that in turn requires accurate estimation of the inter-allele correlation coefficient ρ to distinguish between allele-balanced and allele-imbalanced loci.

3.1 Inter-allele correlation

It can be theoretically shown that the inter-allele correlation coefficient would be a very small real number when it is estimated using allele-balanced loci within a copy number segment. We have experimentally confirmed this expectation on the real datasets. Otherwise, as we have discussed previously, the inter-allele correlation coefficient would be imprecisely estimated and have a negative value when using partially allele-imbalanced loci including copy-neutral LOH.

The difference in inter-allele correlation coefficients between allele-balanced loci and allele-imbalanced loci provides an effective way to separate allele-imbalanced loci (including copy-neutral LOH) from copy-neutral normal segments, based on the associated inter-allele correlation coefficients.

3.2 Identifying copy-neutral loci and genome-wide normalization

In original BACOM and most peer methods, the global mean/median/mode of copy number signal intensities is used for normalizing raw measured copy number signals across experiments or microarrays [4-7,11]. The fundamental assumption for the global mean/median/mode based signal normalization is that copy-neutral component (corresponding to copy number '2') is the most dominant component. However, the dominant component of the intensity mixture distribution rarely coincides with the normal copy number '2' [3]. Our experimental studies on real tumor data confirmed this observation, while also indicated that the largest component(s) often resides within the neighborhood of normal copy number component.

To correctly identify copy-neutral component, we performed sequentially three tasks. First, since the two copy number components (copy numbers '1' and '3') adjacent to the true copy-neutral component (copy number '2') consist of only allele-imbalanced loci, as we have previously illustrated, the associated inter-allele correlation coefficient would have a negative value. We therefore use a narrow-width sliding window centered at a locus to estimate the locus-specific inter-allele correlation coefficient and remove those loci whose correlation coefficients are lower than an automatically-determined threshold value. This step will largely remove most allele-imbalanced loci including copy-neutral LOH. With the remaining allele-balanced loci, the true copy-neutral component is highly probable to be the most dominant component, or well separated enough to be identified, in the revised signal mixture histogram (**Supplementary Figure S2**).

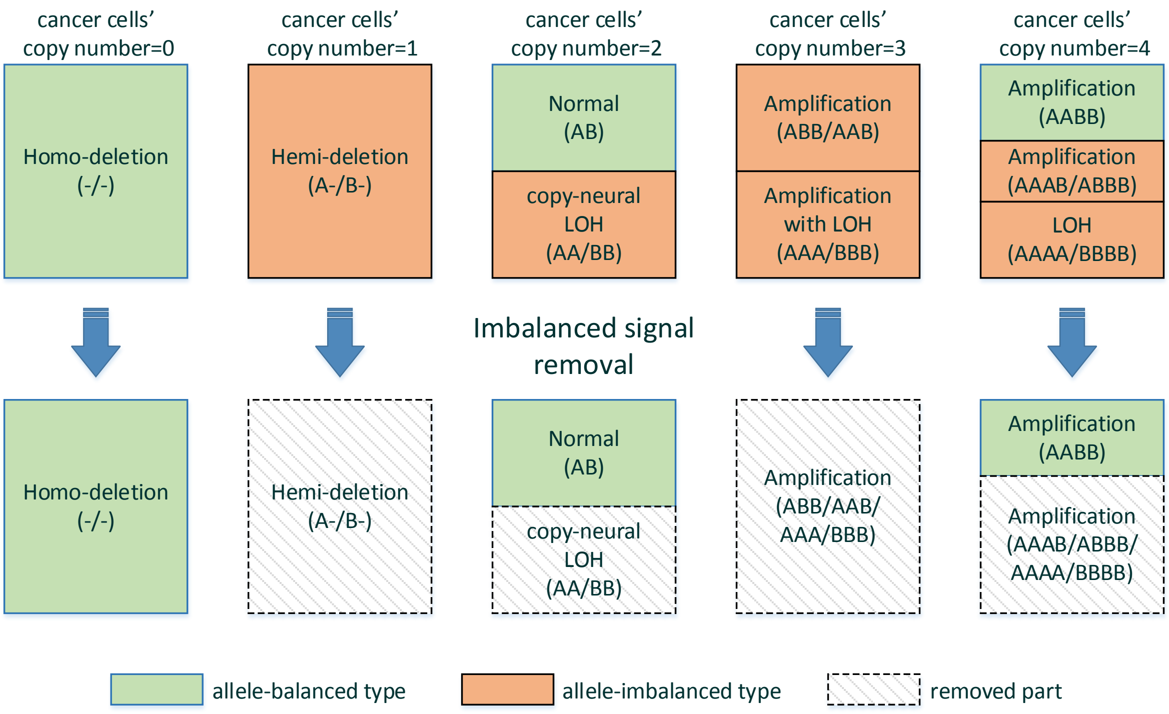

Supplementary Figure S2. Brief illustration of the principles to remove allele-imbalanced loci and revise the signal histogram.

Second, to accurately distinguish between the adjacent components in the signal mixture histogram when the noise level is expected to be significantly high, we replace the raw measured copy number signal at a locus by the averaged value calculated within a narrow-width sliding window centered at the locus. This step will decrease the noise effect, leading to much reduced component variances or a well-separated mixture histogram.

Third, we will identify the copy number component corresponding to '2' and then perform genome-wide absolute normalization. Among the components associated with allele-balanced loci, homo-deletion segments are usually rare and short that can be confirmed by examining real copy number datasets, a sufficiently dominant component while with the lowest mean value will be considered as the normal copy-neutral component.

3.3 Chromosome-wise finer normalization

In real copy number profiling experiments, the hybridization efficiencies vary slightly from one chromosome to another. To adjust these minor signal intensity variations, when the remaining normal copy-neutral loci are sufficient within a chromosome, the mean signal value over these loci (the local baseline) will be calculated and used to perform chromosome-wise finer normalization.

4 Simulation studies

To generate simulation data that have data characteristics similar to real copy number data, we used one real data sample as the ground truth reference, i.e., a pair of matched tumor-normal samples in the TCGA ovarian cancer dataset (TCGA_OV: TCGA-04-1519-01). For the purpose of flexible simulation, we selected chromosome 3 (containing no copy number alteration) as the reference 'template', and used only AB-genotyped probes, consistent with the BACOM methodology [6,7]. To illustrate the imprecise normalization and parameter estimation used in the original BACOM, the simulated raw copy number profile was generated under the following settings: (1) most loci are not copy-neutral but amplified, representing the reality of real copy number profiles in tumor samples; (2) the copy-neutral segments are contaminated with continually distributed copy-neutral LOH loci.

A set of artificial break points were used to divide the chromosome into smaller pieces, each will be later assigned with different copy number statuses, as illustrated in **Supplementary Figure S3**, given below.

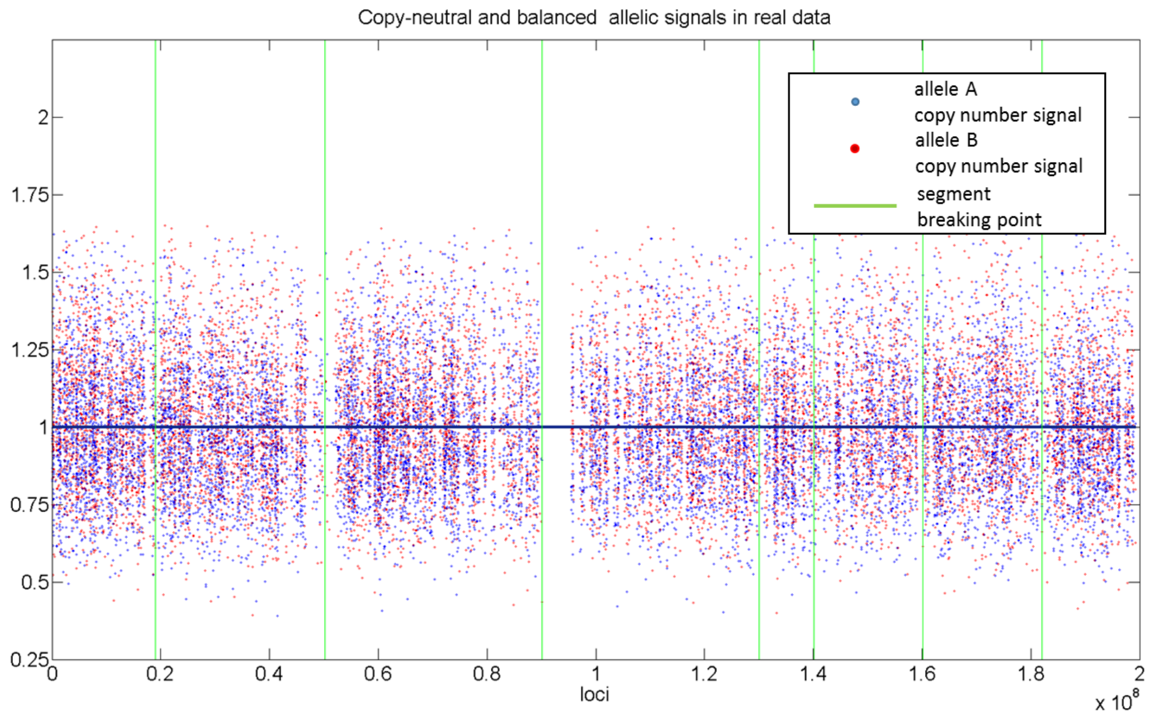

Supplementary Figure S3. Allelic copy number signals from the reference chromosome, superimposed with artificial breaking points.

The chromosome was then broken into segments using the manually generated break points (**Supplementary Figure S4a**); and allelic copy number status was assigned with integers ranging from 0 to 2 for each segment. A preset normal cell fraction α_0 was used to generate the observed copy number signals by mixing the copy number signals of cancer and normal cells. The simulated copy number signals and corresponding allelic signals are shown in **Supplementary Figure S4b** and **Figure 2.**

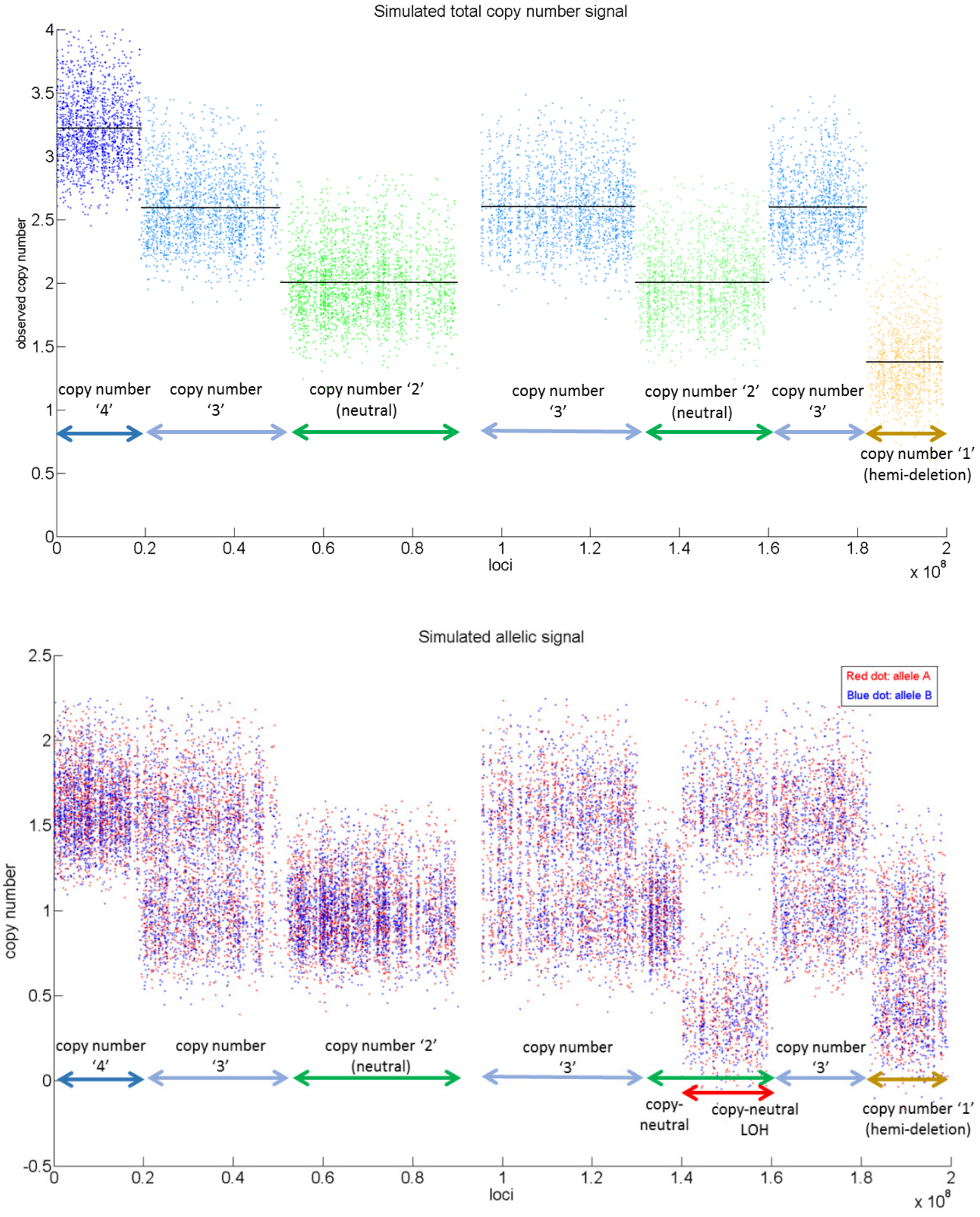

Supplementary Figure S4. Simulated signals of (a) total copy number and (b) allelic copy number, superimposed with artificial breaking points.

We applied both BACOM and BACOM 2.0 to the simulation data. From **Figure 3a**, we can see that the normalization baseline used by the original BACOM does not correspond to the true copy number ‘2’. Owing to a high-level of noise contamination, different copy number components are almost inseparable (significantly overlapped). While from **Figure 3b**, we can see that, after the moving-window average operation is applied to the raw measure signals, adjacent copy number components are well-separated. After eliminating allele-imbalanced loci, the revised signal histogram contains only even and allele-balanced copy number components. The threshold for the inter-allele correlation coefficient for distinguishing allele-balanced and allele-imbalance loci is automatically determined by the valley in the histogram of estimated loci-specific correlation coefficients (**Supplementary Figure S5**).

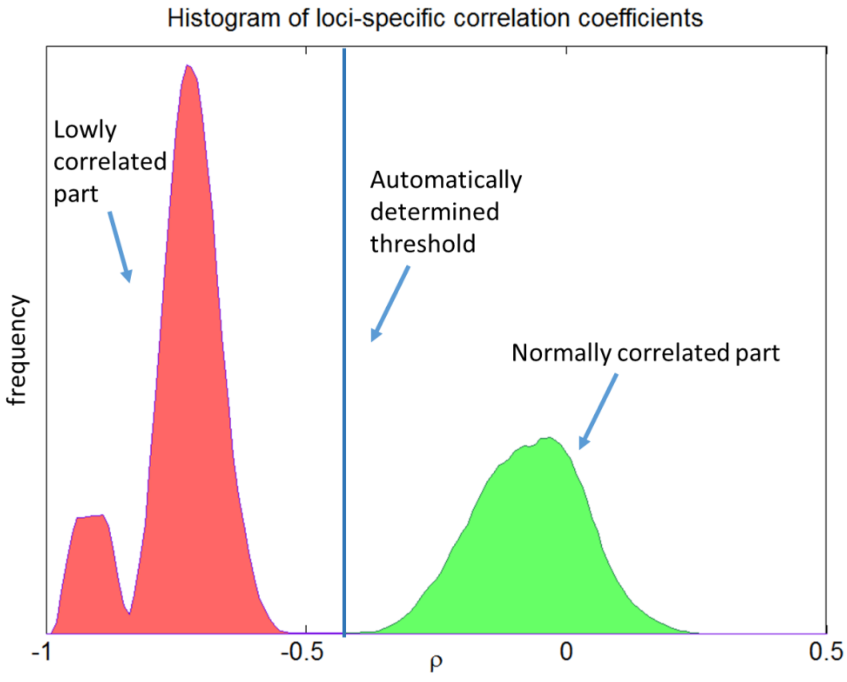

Supplementary Figure S5. Histogram of locus-specific inter-allele correlation coefficients. The allele-imbalanced locus has a lower and negative value of inter-allele correlation coefficient (red), while the allele-balanced locus has a higher value of inter-allele correlation coefficient (green).

From **Supplementary Figure S6**, we can see that, due to imprecise signal normalization in the original BACOM, the copy number segments that actually correspond to copy number ‘3’ are misclassified as the ‘fake’ copy-neutral component, and result in accumulated errors in all subsequent analyses, e.g., biased model parameter estimates,

misclassification of deletions and deletion types. In contrast, BACOM 2.0 has correctly identified the copy number status of all segments, mainly attributed to correct normalization and parameter estimation.

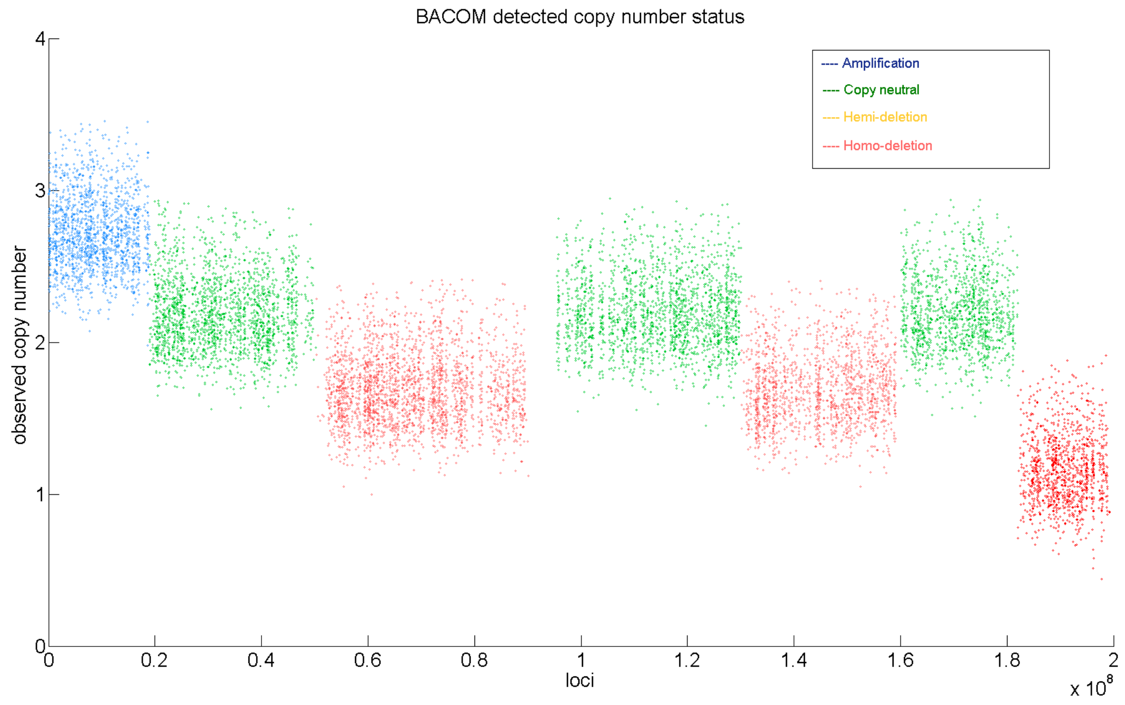

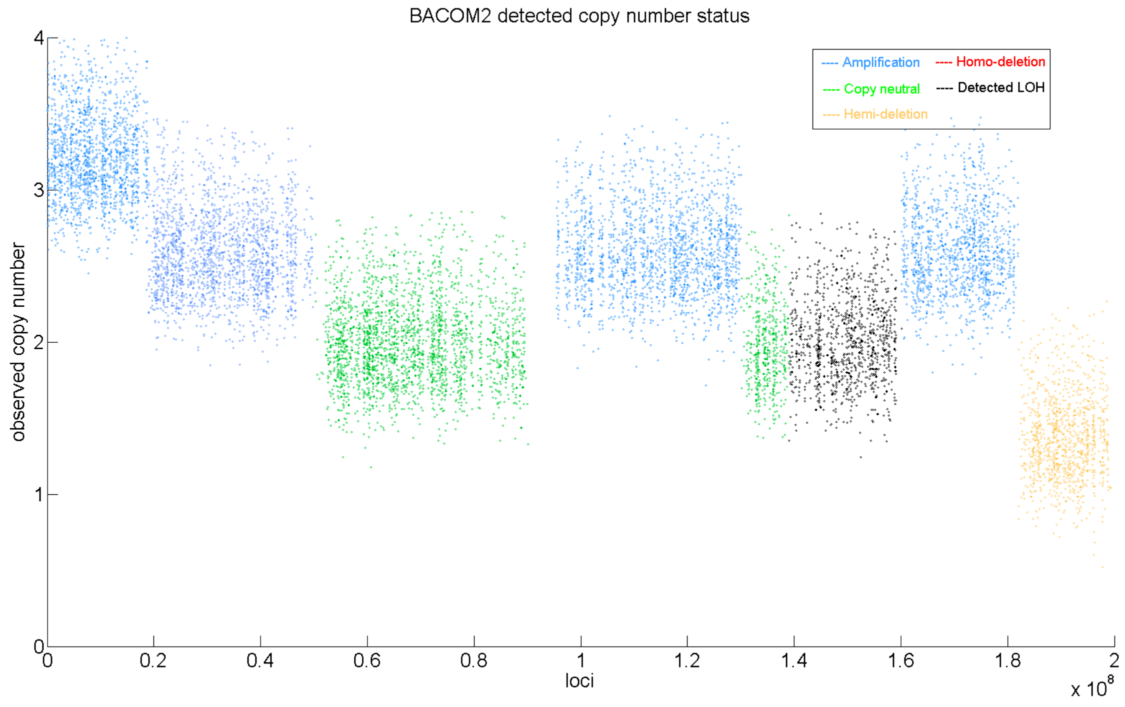

Supplementary Figure S6. Copy number status of segments detected by (a) original BACOM and (b) BACOM 2.0. The copy number signals are accordingly normalized by the two methods.

Comparision between the ground-truth parameter and the estimates made by the original BACOM and (b) BACOM 2.0 (**Table 1**) shows that the original BACOM underestimates the allelic correlation coefficient and overestimates the normal cell fraction, while BACOM 2.0 is able to give a precise estimation.

5 Analyses of real copy number data

The real datasets used in this section are from The Cancer Genome Atlas (TCGA) database, acquired by Affymetrix Genome-Wide Human SNP Array 6.0. In the BACOM 2.0 analysis, we use only matched tumor-normal pairs since somatic alterations are of the interest.

5.1 BACOM 2.0 versus BACOM

Both the original BACOM and BACOM 2.0 are applied to the same datasets, and the estimates of model parameters and sample-wise tumor purity are compared. From **Supplementary Figure S7**, corresponding to the tumor sample TCGA-36-1575-01 (TCGA_OV), we can see that the histograms of raw measured and revised copy number signals show similar characteristics to the simulation data. The normalization baseline was wrongly missed by the original BACOM but accurately identified by BACOM 2.0. As a result, BACOM 2.0 estimated a tumor purity of 0.73, a more realistic estimate, while the original BACOM estimated a tumor purity of only 0.21.

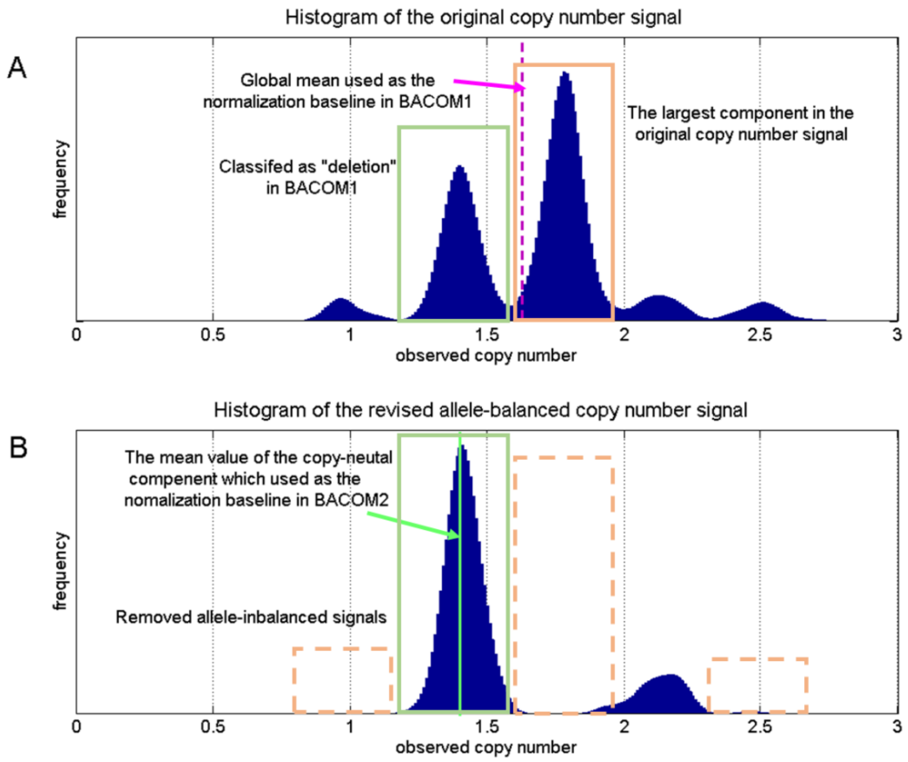

Supplementary Figure S7. (A) Histogram of moving-averaged copy number signals; (B) Histogram of revised copy number signals after eliminating allele-imbalanced loci.

We then applied BACOM 2.0 and BACOM to the samples in the TCGA brain cancer (TCGA_GBM) dataset associated with the tumor sample TCGA-06-0195-01. BACOM 2.0 correctly identified the normalization baseline using the aforementioned detection strategy. Accordingly, BACOM 2.0 estimated a tumor purity of 0.54, while the original BACOM estimated a tumor purity of 0.30. With the estimated tumor purity, we quantified the copy number signals in tumor cells using (6), and estimated an average-ploidy of 3.2 in this sample. From **Supplementary Figure S8**, we can see that, after tumor purity correction, the majority segment means of the copy number profile in tumor cells are near integers. Since the allele-balanced copy number signals (which have never been used in the process of normalizing signals and estimating tumor purity) are also near the expected even integers, we have a compelling form of cross-validation.

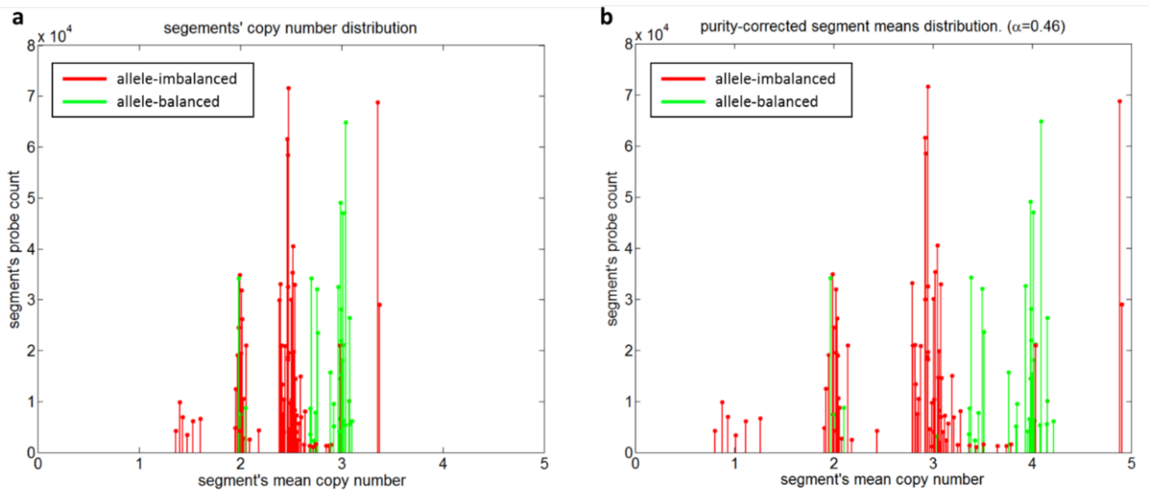

Supplementary Figure S8. Means of copy number segment calculated from (a) normalized signals by BACOM 2.0; and (b) copy number signals in tumor cells, where the height of the bins is the locus counts in the segment.

Next, we analyzed all 466 samples in the TCGA_OV dataset. The average tumor purity estimated by BACOM 2.0 is 0.64, a much more reasonable value than the 0.33 estimated by the original BACOM, see **Figure 3** [4,12,13].

5.2 BACOM 2.0 versus ABSOLUTE

We also compared the results obtained by BACOM2 with the previously reported results obtained by ABSOLUTE [4]. The results released by ABSOLUTE cover two TCGA datasets: ovarian cancer (OV) and brain cancer (GBM). Tested on the same tumor sample as shown in **Supplementary Figure S7**, the average-ploidy estimated by BACOM 2.0 (mean value = 2.33) is very close to the average-ploidy estimated by ABSOLUTE (mean value = 2.73). Sample-wise correlations between the estimates by BACOM 2.0 and ABSOLUTE on the TCGA_OV dataset are given in **Figure 4**, **Supplementary Figure S9**, and **Supplementary Table S1**, and the same comparisons on CGA_GBM dataset are given in **Supplementary Figure S10** and **Supplementary Table S2**.

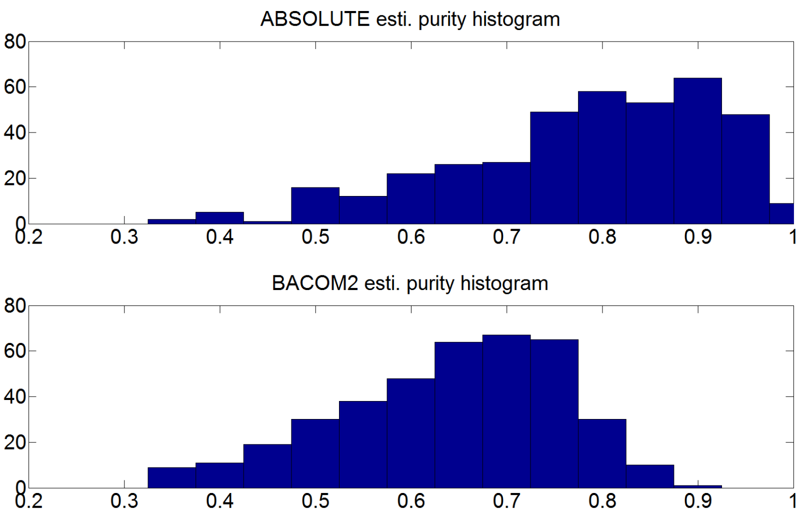

Supplementary Figure S9. Histogram of tumor purity on TCGA_OV dataset estimated by (a) ABSOLUTE and (b) BACOM 2.0

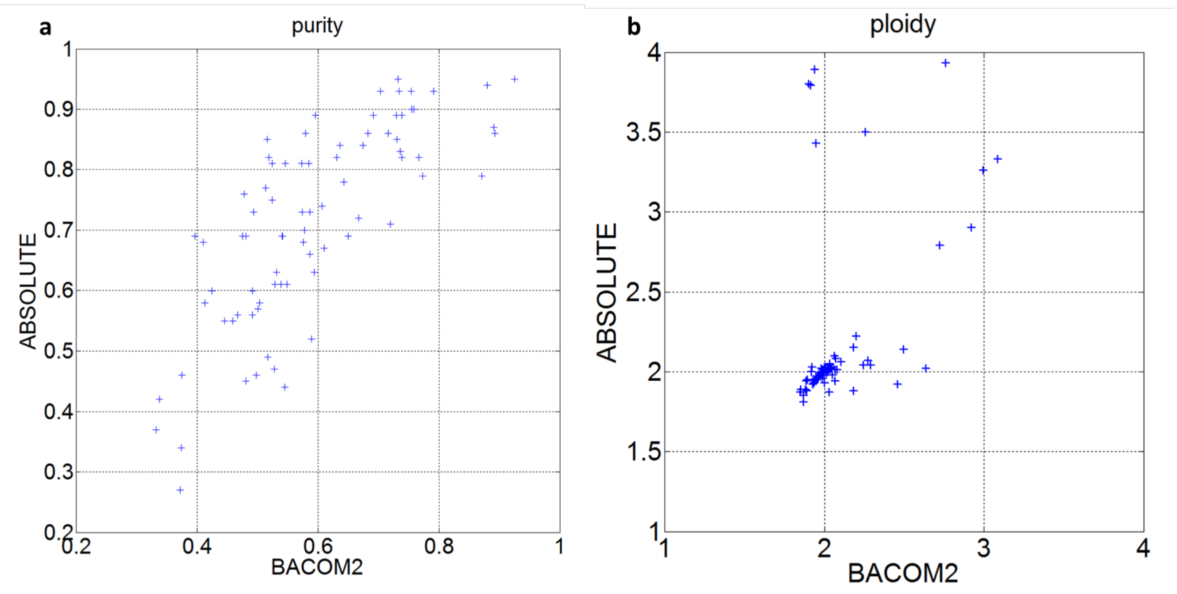

Supplementary Figure S10. Sample-wise comparison between BACOM 2.0 and ABSOLUTE on TCGA brain cancer (TCGA_GBM) samples: (a) Tumor cell purity; (b) Average ploidy.

Supplementary Table S1. Comparasionbetween BACOM 2.0 and ABSOLUTE on TCGA_OV dataset

**TCGA_OV** (n=392)	**BACOM2**	**ABSOLUTE**	**Sample-wise correlation *r***
purity	0.64	0.78	0.74
ploidy	2.33	2.73	0.71

Supplementary Table S2. Comparasionbetween BACOM 2.0 and ABSOLUTE on TCGA_GBM dataset

**TCGA_GBM** (n= 79)	**BACOM2**	**ABSOLUTE**	**Sample-wise correlation *r***
purity	0.59	0.71	0.56
ploidy	2.09	2.17	0.78

5.3 Cross-affirmation by deconvolution on mRNA/protein expression data

In the absence of definite ground truth for the tumor purities in real samples, the validation of a new method for quantifying absolute copy numbers is always problematic. A reasonable alternative is to perform some form of 'cross' affirmation by exploiting the 'orthogonal' information structures provided by the independent sources related to a common set of nature states [14]. We lastly compared the tumor purity estimates by BACOM 2.0 with the estimates by an independent method (called UNDO) that deconvoluted the mixed gene/protein expression profiles of tumor and stromal cells acquired from the same TCGA OV samples [15]. The protein expression data on TCGA ovarian cancer samples were acquired using state-of-the-art mass spectrometry technologies under CPTAC (Clinical Proteomic Tumor Analysis Consortium), to define an integrated proteogenomic landscape and identify factors associated with homologous recombination deficiency (HRD) [16]. These tumor samples were previously characterized by TCGA [17], and key findings were the initial identification of transcriptional

signatures associated with survival and the role of BRCA1/2 and CCNE1 aberrations, and association between HRD and susceptibility to PARP inhibitors and improved survival. The dataset provides quantitative measurements for 10,030 proteins across 122 tumors. To identify a proteomic signature of HRD that could potentially be used to stratify patients for clinical trials [18], tumors were selected for putative HRD as defined by the presence of germline or somatic BRCA1 or BRCA2 mutations, BRCA1 promoter methylation, or homozygous deletion of PTEN [19]. Isobaric peptide labeling (iTRAQ) for quantitation, in conjunction with extensive fractionation and high resolution reversed phase liquid chromatography and high-resolution tandem mass spectrometry, was used for proteomics measurements to provide high depth of coverage for peptide and protein identification. Using the UNDO software, we analyzed the tumor samples with the purity estimate by BACOM 2.0. The experimental result shows that the tumor purity estimates by BACOM 2.0 (based on copy number data) correlates well with the estimates by UNDO (based on gene expression data), consistently achieving a strong average 'cross' correlation coefficient of 0.5~0.6 in multiple runs (**Figure 5b**). We performed the same comparison on the TCGA GBM samples and obtained consistent results (**Supplementary Figure S11**).

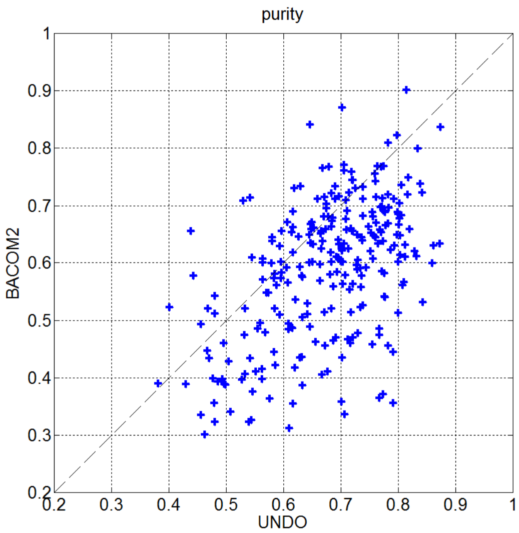

Supplementary Figure S11. Sample-wise correlation (ρ = 0.52) between tumor purity estimated by BACOM 2.0 using copy number data and by UNDO using gene expression data, on the same TCGA_GBM samples. Note that the TCGA-GBM samples have only gene expression data but not protein expression data, thus UNDO was applied to the gene expression data only.

The imperfect 'cross' correlation between the tumor purity estimates made by UNDO, and BACOM 2.0 may be expected and well justified due to the following reasons [15,20]. First, the estimate by UNDO was based on gene expression values, while the estimate by BACOM 2.0 was based on copy number values. Second, though the two information sources are related to a common set of states of nature, they are also 'orthogonal' in various aspects. For example, copy number values are always '2' across all normal cells (e.g., stroma, T-cells, monocytes), while gene expression values are cell type specific. In fact, there are multiple gene expression profiles corresponding to various normal cells. Third, copy number values are generally 'static', while gene expression values are intrinsically 'dynamic'. Such differences can confound the correlation analysis. Fourth, the degree of technical variability, e.g., noise levels, can be significantly different in acquiring copy number versus gene expression signals. For example, in the recent supervised deconvolution work, called ESTIMATE, by Yoshihara et al. [20], in order to obtain a high correlation between the tumor purity estimates derived from copy number and gene expression data, a nonlinear regression function was used to map the 'score' by ESTIMATE [20] to the estimate by ABSOLUTE [4]. Though a higher correlation was obtained and validated on multiple datasets after such nonlinear mapping, it is somewhat 'indirect'.

6 Discussion

In relation to the peer method [4], ABSOLUTE is supported by an elegant yet complex mathematical framework and can select the most likely combination of estimated tumor purity and ploidy by simultaneously estimating purity and ploidy. However, it has been acknowledged that the cornerstone system of equations is underdetermined and various heuristics cannot guarantee a unique and correct solution. Specifically, in the presence of intratumor heterogeneity, the restored copy number signals are not necessarily all integer values, thus using the highest likelihood of producing all integer signals to select the most likely solution may be problematic [21]. For example, in the presence of intratumor heterogeneity, the highest likelihood solution that produces all integer copy numbers can select a solution would be theoretical wrong, since single α corrected signals (removal of normal contamination) should not lead to all integer copy numbers.

In contrast, BACOM 2.0 adopted a divide-and-conquer strategy, i.e., sequentially performs absolute normalization, purity estimation, copy number quantification, and lastly

average-ploidy estimation. In our experimental studies, we have found that some tumor samples exhibit a wide-spread distribution of α values across different segments, consistent with the observation in other studies specifically addressing intratumor heterogeneity [22].

Reference

1 Attiyeh, E. F. *et al.* Genomic copy number determination in cancer cells from single nucleotide polymorphism microarrays based on quantitative genotyping corrected for aneuploidy. *Genome research* **19**, 276-283 (2009).

2 Popova, T. *et al.* Genome Alteration Print (GAP): a tool to visualize and mine complex cancer genomic profiles obtained by SNP arrays. *Genome biology* **10**, R128 (2009).

3 Rasmussen, M. *et al.* Allele-specific copy number analysis of tumor samples with aneuploidy and tumor heterogeneity. *Genome biology* **12**, R108 (2011).

4 Carter, S. L. *et al.* Absolute quantification of somatic DNA alterations in human cancer. *Nature biotechnology* **30**, 413-421 (2012).

5 Wang, Y., Lu, J., Lee, R., Gu, Z. & Clarke, R. Iterative normalization of cDNA microarray data. *IEEE Trans Info Tech Biomed* **6**, 29-37 (2002).

6 Yu, G. *et al.* BACOM: in silico detection of genomic deletion types and correction of normal cell contamination in copy number data. *Bioinformatics* **27**, 1473-1480 (2011).

7 Zhang, B. *et al.* AISAIC: a software suite for accurate identification of significant aberrations in cancers. *Bioinformatics* **30**, 431-433 (2014).

8 Chiang, D. Y. *et al.* High-resolution mapping of copy-number alterations with massively parallel sequencing. *Nature methods* **6**, 99-103 (2009).

9 Hekstra, D., Taussig, A. R., Magnasco, M. & Naef, F. Absolute mRNA concentrations from sequence-specific calibration of oligonucleotide arrays. *Nucleic acids research* **31**, 1962-1968 (2003).

10 Yuan, X. *et al.* Genome-wide identification of significant aberrations in cancer genome. *BMC Genomics* **13**, 342, doi:10.1186/1471-2164-13-342 (2012).

11 Van Loo, P. *et al.* Allele-specific copy number analysis of tumors. *Proc Natl Acad Sci U S A* **107**, 16910-16915 (2010).

12 de Kruijf, E. M. *et al.* Tumor-stroma ratio in the primary tumor is a prognostic factor in early breast cancer patients, especially in triple-negative carcinoma patients. *Breast cancer research and treatment* **125**, 687-696 (2011).

13 Downey, C. L. *et al.* The prognostic significance of tumour-stroma ratio in oestrogen receptor-positive breast cancer. *British journal of cancer* **110**, 1744-1747 (2014).

14 Niv Ahituv, N. & Ronen, B. Orthogonal Information Structures: a Model to Evaluate the Information Provided by a Second Opinion. *Decision Sciences* **19**, 255-268 (1988).

15 Wang, N. *et al.* UNDO: a Bioconductor R package for unsupervised deconvolution of mixed gene expressions in tumor samples. *Bioinformatics* **31**, 137-139 (2015).

16 Zhang, H., Liu, T., Zhang, Z., Payne, S. H. & CPTAC-consortium. Deep proteogenomic characterization of human ovarian cancer. *Nature*, in revision, doi:2014-12-16110 (2015).

17 Cancer Genome Atlas Research, N. Integrated genomic analyses of ovarian carcinoma. *Nature* **474**, 609-615, doi:10.1038/nature10166 (2011).

18 Woodbine, L., Gennery, A. R. & Jeggo, P. A. The clinical impact of deficiency in DNA non-homologous end-joining. *DNA repair* **16**, 84-96, doi:10.1016/j.dnarep.2014.02.011 (2014).

19 McEllin, B. *et al.* PTEN loss compromises homologous recombination repair in astrocytes: implications for glioblastoma therapy with temozolomide or poly(ADP-ribose) polymerase inhibitors. *Cancer research* **70**, 5457-5464, doi:10.1158/0008-5472.CAN-09-4295 (2010).

20 Yoshihara, K. *et al.* Inferring tumour purity and stromal and immune cell admixture from expression data. *Nat Commun* **4**, 2612 (2013).

21 Oesper, L., Mahmoody, A. & Raphael, B. J. THetA: Inferring intra-tumor heterogeneity from high-throughput DNA sequencing data. *Genome biology* **14**, R80 (2013).

22 Yau, C. *et al.* A statistical approach for detecting genomic aberrations in heterogeneous tumor samples from single nucleotide polymorphism genotyping data. *Genome biology* **11**, R92 (2010).
